# Rapid Staining of Circulating Tumor Cells in Three-Dimensional Microwell Dialysis (3D-μDialysis) Chip

**DOI:** 10.1038/s41598-017-09829-3

**Published:** 2017-09-12

**Authors:** Wanying Cho, Rangadhar Pradhan, Hsin Ying Chen, Yi-Hsuan Weng, Hsueh Yao Chu, Fan-Gang Tseng, Chien-Ping Lin, Jeng-Kai Jiang

**Affiliations:** 1 Department of Engineering and System Science, Frontier Research Center on Fundamental and Applied Sciences of Matters, National Tsing-Hua University, Hsinchu, Taiwan, ROC; 2 Institute of NanoEngineering and MicroSystems, National Tsing-Hua University, Hsinchu, Taiwan, ROC; 3 Division of Colorectal Surgery, Department of Surgery, Veterans General Hospital, Taipei, Taiwan, ROC; 4 Research Center for Applied Sciences, Academia Sinica, Taipei, Taiwan, ROC

## Abstract

The conventional techniques to detect circulating tumour cells (CTCs) are lengthy and the use of centrifugal forces in this technique may cause cell mortality. As the number of CTCs in patients is quite low, the present study aims towards a gentler diagnostic procedure so as not to lose too many CTCs during the sample preparation process. Hence, a Three-Dimensional Microwell dialysis (3D-μDialysis) chip was designed in this study to perform gentle fluorescence-removal process by using dialysis-type flow processes without centrifuging. This leads to a minimum manual handling of CTCs obtained in our study without any contamination. In addition, a rapid staining process which necessitates only about half the time of conventional techniques (35 minutes instead of 90 minutes) is being illustrated by the employment of dialysis process (by dynamically removing water and waste at once) instead of only static diffusion (by statically removing only waste by diffusion). Staining efficiency of our technique is improved over conventional staining because of the flow rate in 3D-μDialysis staining. Moreover, the staining process has been validated with clinical whole blood samples from three TNM stage IV colon cancer patients. The current technique may be termed as “miniature rapid staining and dialysing system”.

## Introduction

Cancer is the leading cause of death globally and a burden upon society across the world. It is a potentially abysmal cell growth which is associated with exposure to ionizing radiations, environmental pollutants, certain infections and unhealthy lifestyle. It is the leading cause of death in economically developing countries and second leading cause of death in economically developed countries according to the world health organization update of 2008. It is being estimated that 15 million cases and 10 million new deaths are to occur in the next five years^1^. The most common forms of cancers are colon, lung, liver breast and oral followed by prostate, thyroid, skin, and oesophageal ones. Much still remains to be known about several major types of cancer. The identification of predictive factors of cancer is important for patients and physicians to choose from adequate treatment facilities available.

Metastases is the factor which is potentially life threatening in development of a malignancy. It may be located in the different areas of same organ or different organs and its anatomical establishment plays a huge role in response to the conventional therapy^2^. Efficiently, tumour cells travel via the bloodstream and then extravagate the bloodstream to infringe the novel tissue^3^ and invade into the parenchyma of foreign tissue to acclimatize and reproduce to form metastases. Because successful dissemination mostly occurs through blood, circulating tumor cells (CTCs) that have been shed in the vasculature evoke a lot of interest. Numerous studies in the past have shown that CTCs may be used as a biomarker to predict malady advancement and mode of treatment in metastatic^4^ and probably even in first-stage cancer patients^5^. Studies have also depicted that the amount of CTCs have a positive correlation to survival rate and disease prediction of patients^6^, so the detection and enumeration of CTCs is very important for the treatment of cancer. One of the most self-evident implications of CTCs is that they are minimally invasive indicators^7^. Detection of CTCs can reveal mint of information rather than just the presence of a tumor.

In order to effectively cure the cancer, the basics of CTCs including isolation, detection, characterization, and molecular mechanism of their circulation in the blood stream which is determined with the primary tumor correlations, contribute to the choice of treatment and prognosis. The major limitation in isolation of CTCs is their availability in limited numbers, only about 1 to 10 CTCs per millilitre in whole blood of patients. In other words, about millions of white blood cells have one CTC (10^6^~10^7^: 1); or billions of red blood cells will have one CTC (10^9^~10^10^: 1).

As the number of CTCs is quite few, the need of the hour is a gentler way of sample preparation process in order not to lose CTCs. In the traditional way of staining process by mixing and centrifugation procedures^8–10^, a lengthy protocol (1.5 hours for 3 dyes) is usually required as well as exposure to centrifugation force which may be harmful for cell integrity and viability. There is yet lot of scope for improvisation in the quantitative as well as qualitative aspects of tumour cell detection.

Borrowing from the technological merits of microfluidic technology, several microfluidic systems have been proposed for the isolation, staining and characterization of CTCs with higher success measures compared to the predictable macro-scale devices^11,12^. For example, the CTC-iChip^13^, lateral magnetophoresis chip^14^, two-stage microfluidic chip^15^, nanostructure embedded microchips^16^, parallel flow micro-aperture chip^17^, and the herringbone chip^18^ mainly make use of EpCAM- or other surface antigen-specific antibodies to identify and arrest CTCs in the microfluidic systems. On a whole, these systems have been confirmed to segregate CTCs with high CTC purity (14–70%)^13,15,18^ and extraordinary recovery rate (77–91.8%)^13,16,18^.

Even though the above mentioned positive selection-based CTC isolation schemes (either the conventional- or microfluidic-based methods) have been technically established to segregate and purify CTCs, there are some significant biological concerns that should be additionally considered like the staining procedures which shouldn’t be lengthy enough for cell to lose its integrity and viability. Although recent advances have allowed the procedure to be carried out with minimal loss of cell types^19^, the process of immunofluorescence staining of cells requires centrifugal force to wash residues, which is not only time consuming but also harmful for cells. To overcome this concern and speed up the staining process, a 3D Microwell Dialysis (3D-μDialysis) chip with a porous PDMS membrane is designed in this study to perform gentle fluorescence-removal process by a dialysis-type diffusion process on CTCs and WBCs without centrifugation. Through a gentle dialysis process, this device can reduce staining process time from 1–1.5 hours down to 35 minutes and still ensure high cell viability.

## Results

### Working mechanism of 3D-μDialysis chip

**Figure 1:**
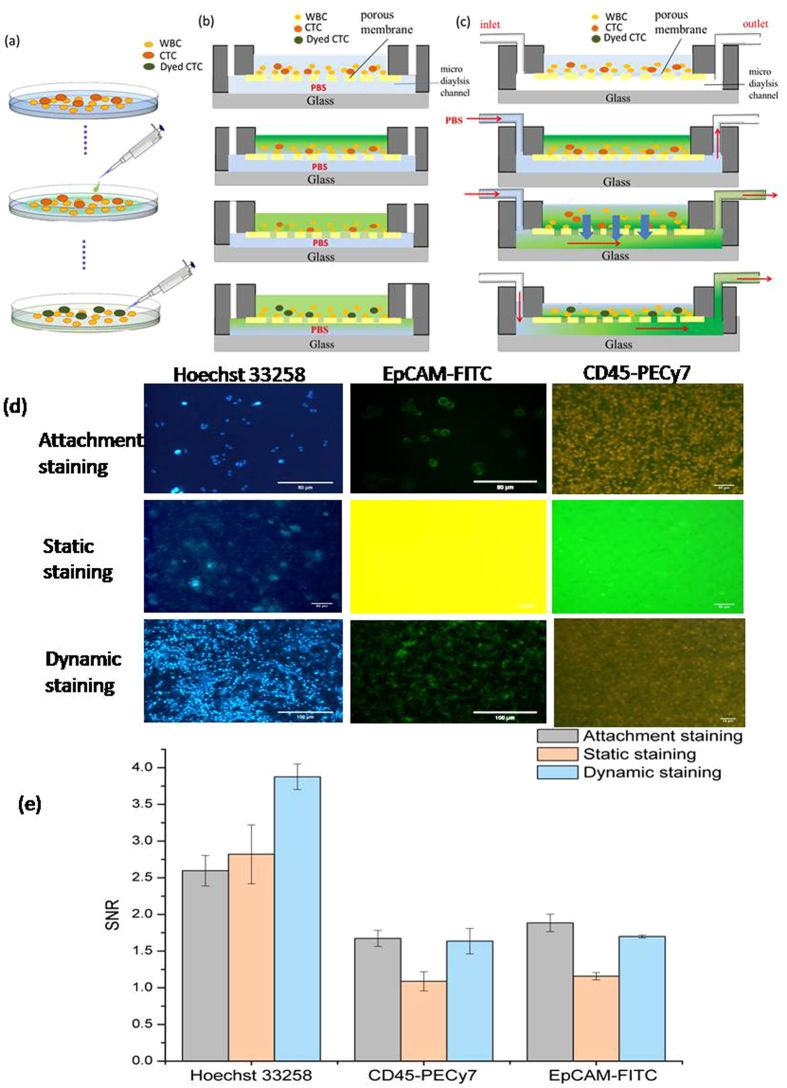
The schematic principle of (**a**) attachment staining (**b**) static staining, and (**c**) dynamic staining along with the (**d**) fluorescence staining results and (**e**) signal to noise ratio (SNR) by three different staining methods.

The working mechanism of attachment staining and static, along with dynamic staining using 3D-μDialysis chip is schematically described in Fig. 1(a–c) respectively. The attachment staining can be defined as the conventional staining carried out by using petridish and spreading the stains on the top of the surfaces. The static and dynamic staining is carried out by using 3D-μDialysis chip at no flow and at a certain flow respectively. In the actual experiment, cell suspension with 500,000 cells inclusive of white blood cells and CTCs are inserted into the top open-chamber and then a syringe pump is utilized to inject PBS solution into the bottom micro dialysis channel of the chip, while the PDMS porous membrane was sandwiched between the top chamber and the bottom micro dialysis channel to accommodate the cells without losing yet allow fluorescence dye to diffuse downward. The WBCs and CTCs descend into a dense array with multiple layers (3–10) on the top of the PDMS porous membrane for in-parallel staining. Dye conjugated antibodies were then introduced from top of the open milichamber and closely stain antibodies on individual cell surfaces in-parallel. When PBS solution is forcing through the bottom micro dialysis channel, a high concentration gradient of the fluorescence dye would be built up between the top open chamber and the bottom micro dialysis channel, and the residual solution is dialyzed out from the top chamber to the bottom micro dialysis channel by dialysis type diffusion (removing water and waste simultaneously, Fig. 1c) instead of static diffusion (removing waste partially, Fig. 1b) process. Thus static staining does not wash out the unused dyes such as Hoechst, EpCAM-FITC and CD45-PECy7 completely from the staining chamber. As Hoechst only stains DNA, its residual does not affect the system and produces normal image under a microscope. However, the residuals of EpCAM-FITC and CD45-PECy7 produce plain yellow and green as shown in Fig. 1b under a microscope by following their corresponding staining properties. By employing this gentle dilution method, cells would be stained uniformly and rapidly within 30 minutes for 3 different fluorescence dyes successively without harmful fluidic forces exerted on cells. The results will also be compared with the traditional attachment staining shown in Fig. 1a.

### 3D-μDialysis chip staining Tests

#### Attachment, static, and dynamic staining

For traditional attachment staining test, the SNR is 2.59 ± 0.21, 1.67 ± 0.11, and 1.88 ± 0.03 at 30 minute for Hoechst, CD45-PECy7, and EpCAM-FITC respectively as depicted in Fig. 1. In static staining (zero flow rate in micro dialysis channel), the gradients of fluorescence drive the dyes to diffuse slowly across the membrane and the images are illustrated in Fig. S1. The SNR values of static staining are 2.82 ± 0.40, 1.08 ± 0.13 and 1.15 ± 0.49 at 10 minute for Hoechst, CD45-PECy7, and EpCAM-FITC respectively while the individual SNR values for dynamic staining are 3.88 ± 0.17, 1.64 ± 0.17 and 1.70 ± 0.02. The results show that 3D-μDialysis chip has equal staining effect as conventional attachment staining process in a limited time span and without an optimized flow. The small differences can be omitted as cell viability and impurities in blood affect the light intensity. For a better comparison between conventional staining and 3D-μDialysis, cell viability test was carried out by using propidium bromide (PI) dye in both the cases and the result was described in Fig. S2. The numbers of healthy cells (10 to 18 µm size) are 871 ± 28 and 695 ± 10 for sample without centrifugation and with centrifugation respectively. Thus it can be inferred from the Fig. S2 that the cell count decreases 20.20 ± 2.8% after going through centrifugation. As cells are ruptured by centrifugation the PI dye enters into the cell and stains DNA while it cannot penetrate the cell membrane of healthy cells stained by 3D-μDialysis chip. Thus the PI fluorescence is brighter in case of centrifuged cells which infers the cell death due to centrifugation. The cells collected from centrifugation are round in shape as they are ruptured due to centrifugation. Also the irregular shaped cells stained by PI found in case of sample without centrifugation which may represent the unhealthy cells originally existed in the suspension.

### Optimization of flow rate and time individual test for 3 fluorescence dyes

Figure S3 represents one of a typical histogram of static and dynamic staining for Hoechst 33258 at a flow rate of 10 ml/hr. In static staining, the peak of background and noise is not easy to differentiate leading to a poor SNR. However, the peak of background and noise can be differentiated clearly in dynamic staining. This may be attributed to the flow of PBS in the micro dialysis channel leading to a better staining over static one.

**Figure 2:**
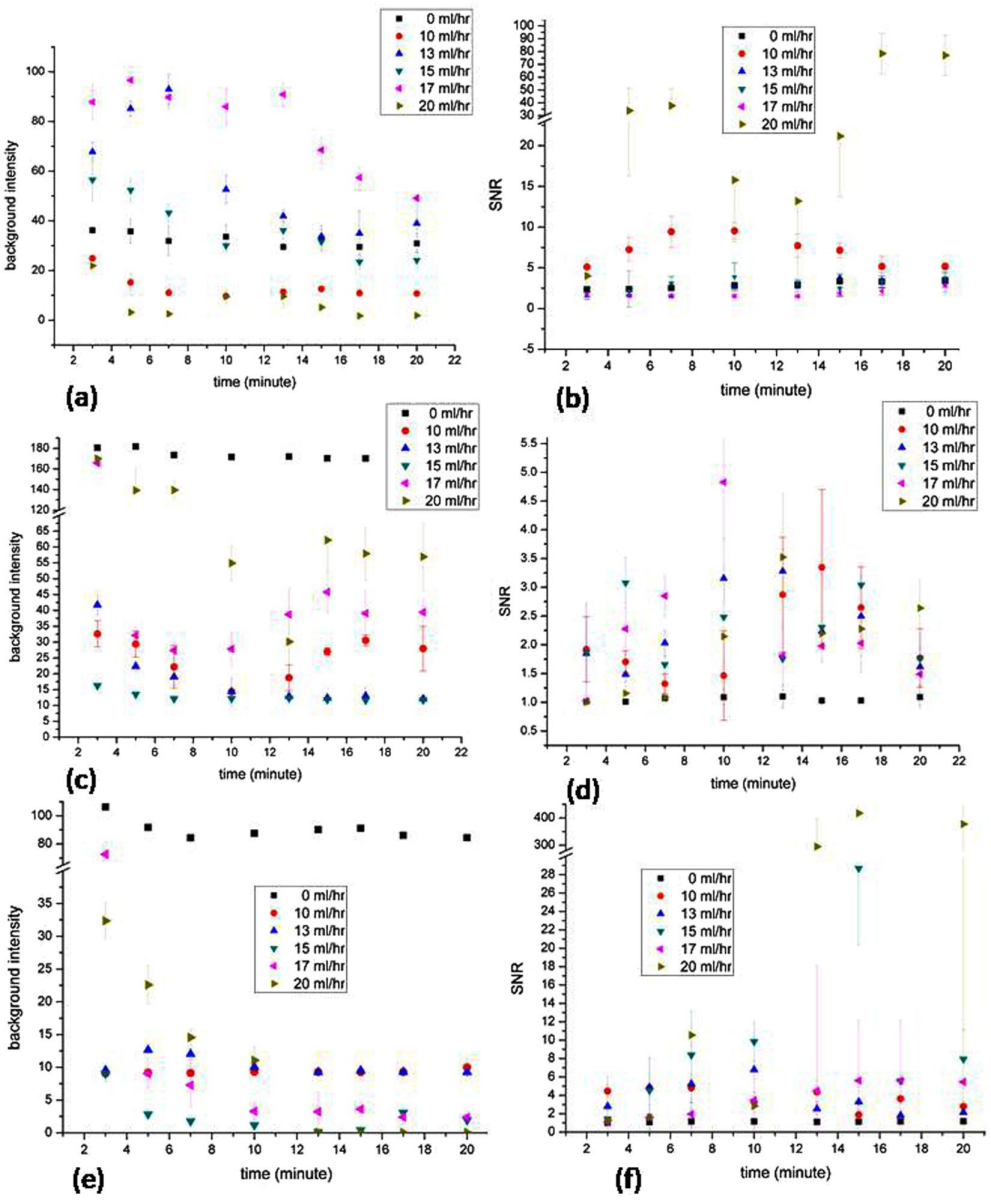
The time course of staining progress for three different dyes, (**a**) background and (**b**) SNR of Hoehest 33258, (**c**) background and (**d**) SNR of CD45-PECy7, and (**e**) background and (**f**) SNR of EpCAM-FITC.

Figure 2(a),(c),(e) represents the background intensity with time for Hoechst, CD45-PECy7, and EpCAM-FITC dynamic staining at a flow rate from 0–20 ml/hr, respectively. Due to the PBS flow in the micro dialysis channel, the background intensity in each experiment group decreases with the increase of time. At certain time, the floating impurities in the blood would make some varieties in the staining process and the numerical data goes high. Hence we focus on the tendency curve on each experiment groups. Considering different fluorescence dyes, the Hoechest 33258 and EpCAM-FITC staining show good results as the background light intensities approach to zero in the last half of the staining process. Figure 2(b,d,f) represents the SNR with time for Hoechst, CD45-PECy7, and EpCAM-FITC respectively. Both the light intensity decreases with the continuous PBS flow and the SNR data is strongly affected by the background light intensity. Once the background light intensity goes under 1.0, the SNR will soar high, and in Fig. 2(f), the SNR at 13^th^ minute of EpCAM-FITC is above 294.68 ± 104.32. This enhanced error may be due to brighter fluorescence contributed by cell as well as clear background light intensity which is 0.09 ± 0.006 at 13^th^ minute of EpCAM-FITC.

From the above data it is found that steady staining curve is present in the first half of the staining process, so a most suitable time is chosen as 10 minute for 3 fluorescence dyes. Considering the staining effect at 10 minute for Hoechst, CD45-PECy7, and EpCAM-FITC, the flow rate is optimized as 10 ml/hr for Hoechest and 15 ml/hr for both CD45-PECy7 and EpCAM-FITC.

**Figure 3:**
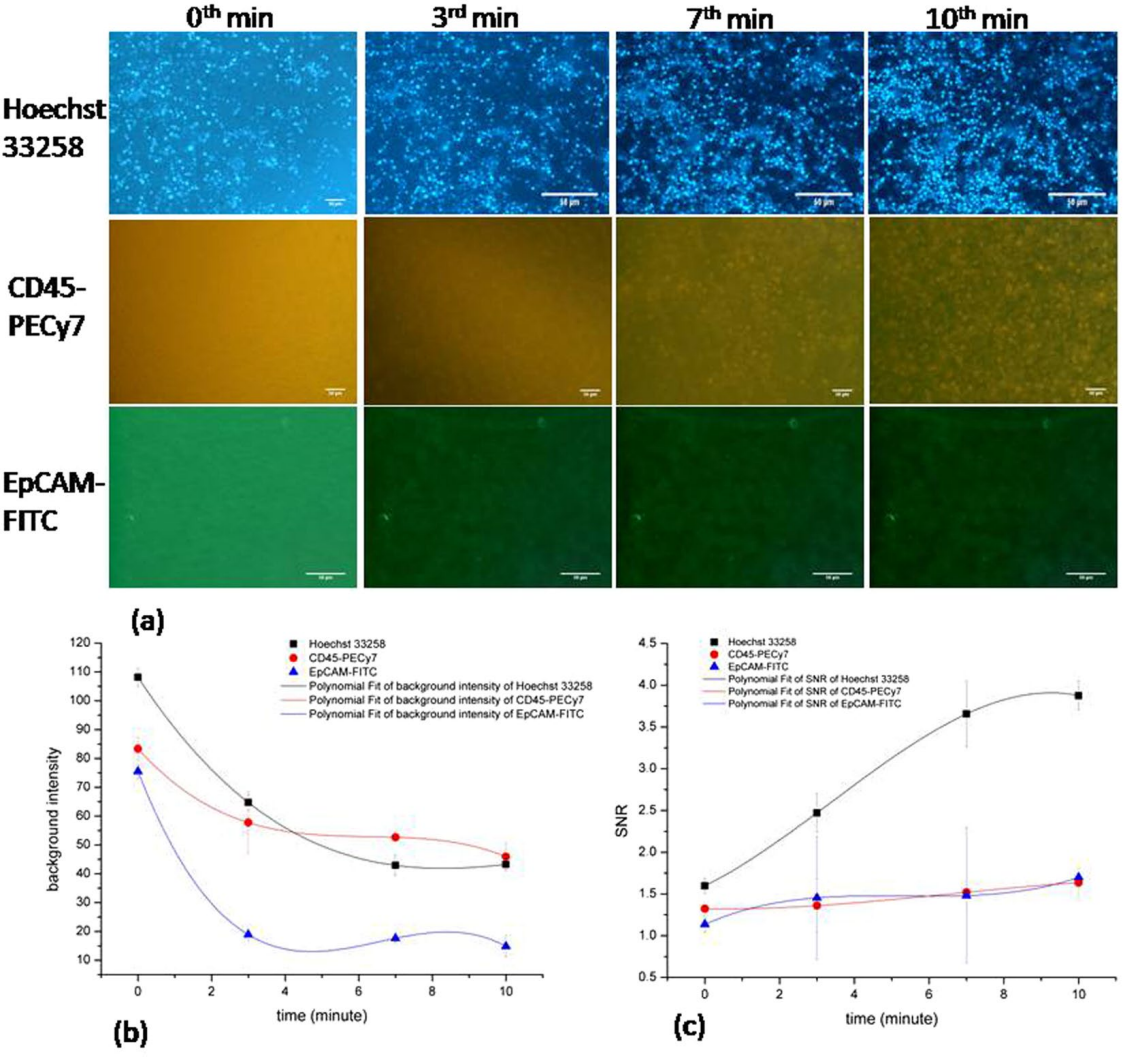
Time course of individual dynamic staining of three different dyes on BT474 cells (Hoechst 33258 and EpCAM-FITC) and WBCs (CD45-PECy7) in 3D-µDialysis chip with optimized conditions. (**a**) Images of staining as it progress (**b**) time course of background noise, and (**c**) SNR.

Figure 3 describes the dynamic staining for different dyes by using the optimized flow rate and time. From the figure it is evident that both the background light intensity and SNR can be fit in a smooth curve, as shown in Fig. 3(b,c). The SNR is 3.88 ± 0.17, 1.64 ± 0.17 and 1.70 ± 0.02 at 10 minute for Hoechst, CD45-PECy7, and EpCAM-FITC respectively.

### Mixed staining test

**Figure 4:**
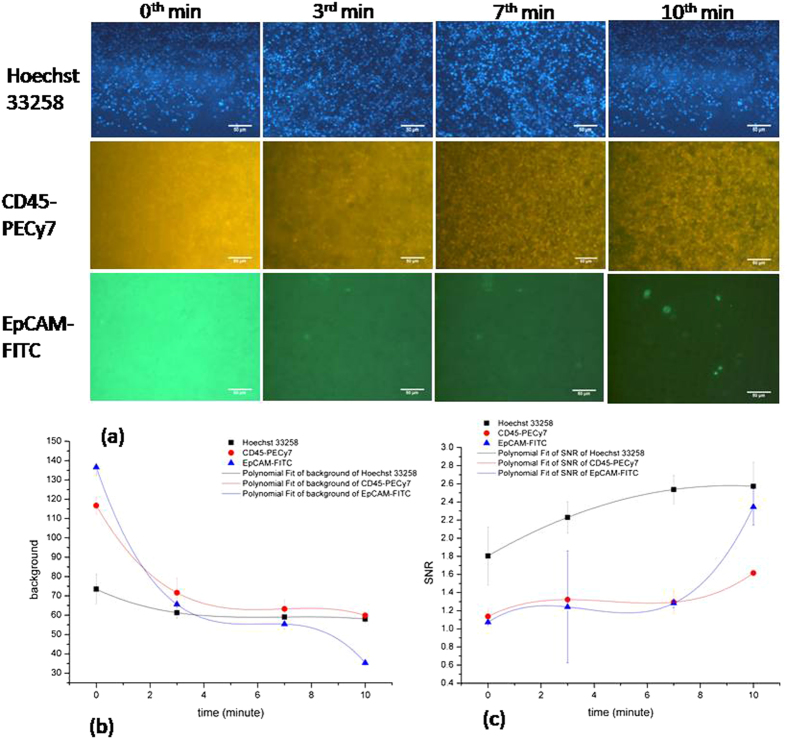
Time course of continuous dynamic staining of three dyes on a mixture of BT474 cells and WBCs in 3D-µDialysis chip. (**a**) Images of staining progress (**b**) time course of background noise, and (**c**) SNR. Each scale bar is 50 µm

Figure 4 depicts the 3 mixed dyes staining results which infer a good staining of individual dyes in a mixed environment. The background noise intensity decreases with increases of time while the signal to noise ratio increases with the time as shown in individual dye staining in ten minutes. In Fig. 4(b), the curve in background and SNR match the same type with the individual staining test. Also the total time consumption in staining for 3D-µDialysis staining is less than (35 min) the traditional staining (90 min) process as described in Fig. S4.

### Clinical sample test

**Figure 5:**
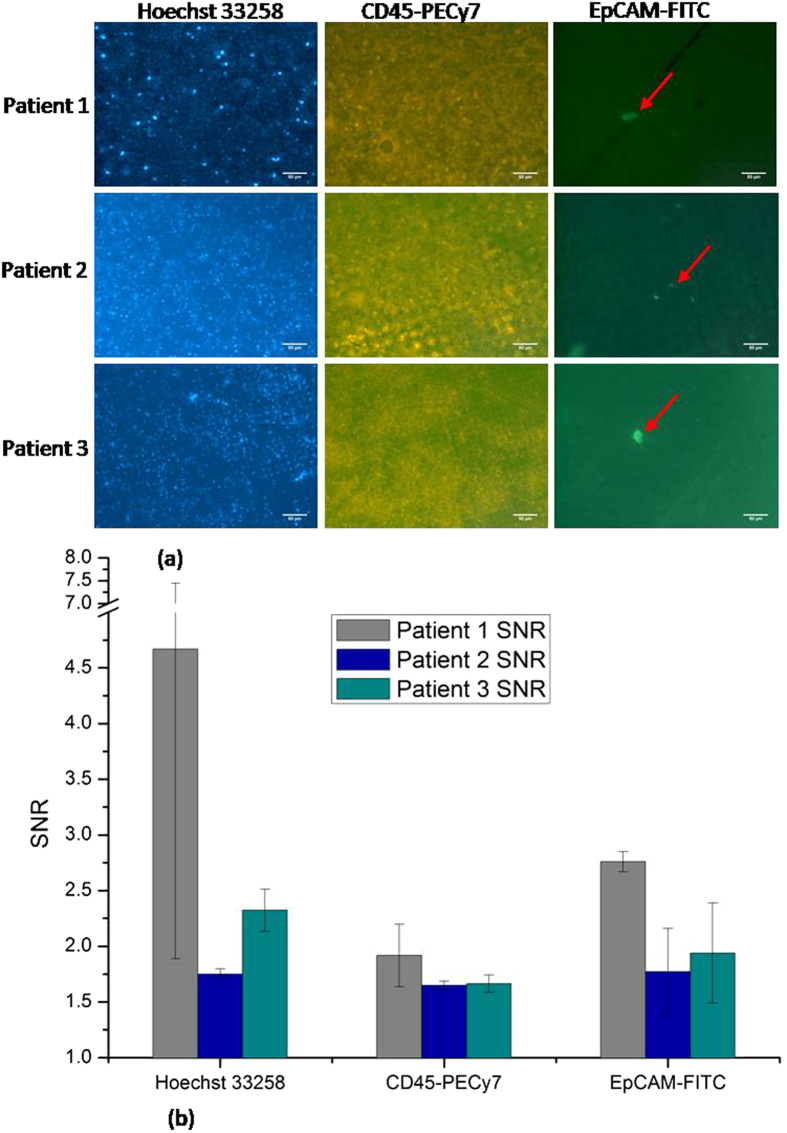
Continuous dynamic staining of three dyes for three patients’ bloods containing colon cancer CTCs in 3D-µDialysis chip. (**a**) Images of staining results, and (**b**) the SNR and deviation of analysis. (The arrow indicates the CTCs).

Recently, an experiment was carried out by collaborating with Taipei Veterans General Hospital for detection of stage IV colon cancer by using our fabricated μDialysis chip and is illustrated in Fig. 5. Three cases have been received and have been treated with the above cited staining procedure by using μDialysis chip. The procedures were completed within 24 hours of collection of blood samples. There were about 500,000 cells stained on μDialysis chip. The arrows in the Fig. 5 show the CTCs which define that all the three staining procedures by using μDialysis chip show good result for detecting colon cancer CTCs, with a EpCam signal to background noise ratio above 2.16 ± 0.31, suitable for clinical applications.

### Staining comparison by using COMSOL Multiphysics

The comparison of conventional and 3D-μDialysis staining was carried out by using COMSOL Multiphysics and the results are represented in Fig. S5. The highest and lowest concentration values are 1.8 × 10^−2^ and 1.42 × 10^−2^ mmol/m^3^ respectively in case of dynamic staining for an applied concentration of 1.8 × 10^−2^ mmol/m^3^ on the top surface. However such values in case of conventional staining are 1.8 × 10^−2^ and 1.93 × 10^−4^ mmol/m^3^ respectively. Thus it can be inferred that the distribution of concentration of dye is better in flow condition (3D-μDialysis chip) as compared to system without flow (conventional staining). The concentration of the dye in the dynamic staining process was distributed evenly as compared to the static staining case as the diffusion takes place consistently in case of dynamic staining. This may be due to the excellent flow driven staining which is present in case of μDialysis chip staining and absent in attachment staining. This result can be compared to the practical experimentation as described above. The qualitative and quantitative covenant amongst the computed and measured results authenticates the simulation.

## Discussion

CTCs have the inherent property to detach themselves from parent cell and start acting like a free flowing unit. Therefore the isolation, identification, and characterization of the CTCs in blood are prerequisite for determination of CTCs as biomarkers. The need of the hour is the proficiency to obtain CTCs from blood samples of patients to facilitate treatment objectives on tumor cells in real time. The available standard for CTC enumeration is the Cell Search system^20^ which has its own advantages and disadvantages. The technique along with offering better control of the microenvironment during isolation and staining of CTCs, encourages amalgamation and mechanization for high throughput sample processing. But the microfluidics based technologies are all based on high dominant drag forces which enable the laminar flow to guide the cellular particles in a streamline. It is also evident that numerous microfluidic technologies as discussed earlier have been developed across the globe but insufficient information has been obtained regarding their validation with authentic clinical samples. Also the study by Hou *et al*.^21^ shows that EpCAM-based microfluidic technologies are predominantly advantageous for CTCs enumeration due to high capture purity.

Development of this 3D-μDialysis chip is a singularly significant bioengineering challenge which required in-depth analysis and optimization of system properties. The system properties are being categorized into two parts, system device and system fabrication. The important design and fabrications which were kept in mind are the biocompatibility considerations of the cells, their sterilization, the 3D geometry of the cells and the surface chemistry. The foremost advantage of this 3D-μDialysis chip is minimal manual handling of the CTCs (1–85 cells/ml obtained in our study) during the isolation and immunostaining steps done on-chip to avoid contamination with a small imaging area to facilitate counting. Dynamic staining on the 3D-μDialysis chip is another path-breaking step as it helps to stain CTCs effectively as compared to conventional staining. The dynamic staining stains maximum CTCs because in static staining process the flow rate was 0 ml/hr whereas that in dynamic staining was optimized at 15 ml/hr of the two protein dyes (CD45-PECy7 and EpCAM-FITC). Furthermore, there was a continuous concentration-gradient of fluorescence dyes between the top open milichamber and the bottom micro dialysis channel in dynamic staining whereas in static staining the residual dyes were diffused in a fixed area. As a result, the dyes in the dynamic staining has more chance to encounter with each cell through the flow action, while static staining allows only a small portion of dyes encountering with cells at worse distribution uniformity. This would lead to fewer CTCs stained in the static staining process. On the other hand, in the no-flow condition of attachment staining process, in addition to the aforementioned mechanism, individual cancer cells would form highly localized pockets surrounded by WBCs at the bottom of flasks and expose only a small portion of surface area to solution, hence very few cells can be stained.

The staining effect of the dyes improved in the 3D-μDialysis chip as may be viewed from the SNR of the individual dyes staining. The SNR is a straightforward calculation that estimates the optimal specificity and fluorescence intensity of the samples. The optimal antibody concentration produces the maximum signal to noise ratio. The SNRs of individual dyes are equal to that of traditional attachment staining in EpCAM-FITC and CD45-PECy7 and was better than that of traditional attachment staining in Hoechst 33258. This proves that the improved 3D-μDialysis staining is due to the reduction in undesired non-specific or background staining and the increased flow rate of the dialysis solutions (Hoechst33258 with a flow speed of 10 ml/hr; EpCAM-FITC and CD45-PECy7 with a speed of 15 mL/hr). The time and flow driven staining allowed the individual fluorescent dyes sufficiently to bind with the epitope.

In case of individual staining for 3 fluorescence dyes, the cell suspension interacts with the PBS dialysis flow and form cell clusters easily. Hence the fluorescence image only focused on one of the layers in the staining region. According to the fluorescence image analysis, the light intensity increases with the death of the cells. Thus it is not precise to judge the SNR, when the background light intensity approaches towards zero after 10 minutes of staining time. As the stability of immunofluorescence varies in a big range as observed from the analysis of numerical data of 3D-μDialysis staining, it is obvious to discuss the background intensity after 10 minutes. Thus the best time to finish the staining process is optimized at 10 minute.

The total staining time was reduced from 90 minutes (traditional staining) to 35 minutes (3D-μDialysis staining). This successfully renders less exposure of the CTCs to the continuous shear and ensures minimum detrimental shear-related changes to the CTCs phenotype which is of paramount importance. The sample preparation is also minimal in this case as it does not require expensive devices.

These results were validated using COMSOL Multiphysics which shows that the μDialysis chip staining is better than the conventional staining. The diffusion process was understood by carrying out the simulation using a dye in very low concentration.

Our team anticipates that such user friendly and integrated enabling device may attract the attention of large biopharmaceutical companies and academic institution which will support to reduce the disease diagnostics based expenditure and time. This may particularly attract clinical and diagnostic laboratories as they allow rapid staining of small amount of patient derived cells too. The reduced scale and cost^22^ of this device negates the need of super specialty laboratories and cohesive instrumentation and combined staining results in much shorter assay times.

## Conclusion

The current technique may be termed as “miniature rapid staining and dialysing system”. To this end, our team believes that our staining technique on a 3D-μDialysis chip offers unique advantages over other conventional staining techniques by improving signal to noise ratio effectively. In addition to BT474 cell-line and HeLa cell-line, clinical whole blood samples from three TNM stage IV colon cancer patients were also successfully stained in 35 minutes with mixed stains such as Hoechst, EpCAM-FITC and CD45-PECy7 by using this 3D-μDialysis chip after pre-screening out erythrocytes, and the result shows a decent 2.16 signal to noise ratio of EpCAM-FITC suggesting a promising tool for utilization in real clinical applications. This is a very versatile and convenient technique for the separation and fast staining of the CTCs and also an example of new age microfluidic technology that may probably support the development of new therapeutic approaches.

## Methods

### Materials and reagents

Silicon wafers were purchased from Semiconductor Wafer limited, Taiwan. SU8 was obtained from MicroChem. HeLa cell line was purchased from American Type Cell Culture (ATTC). DMEM, RPMI, FBS were supplied by Invitrogen, Taiwan. All other chemical used in the present experiments were obtained from Sigma Aldrich, Taiwan.

### Design of the microfluidic chip and its functionalization

**Figure 6:**
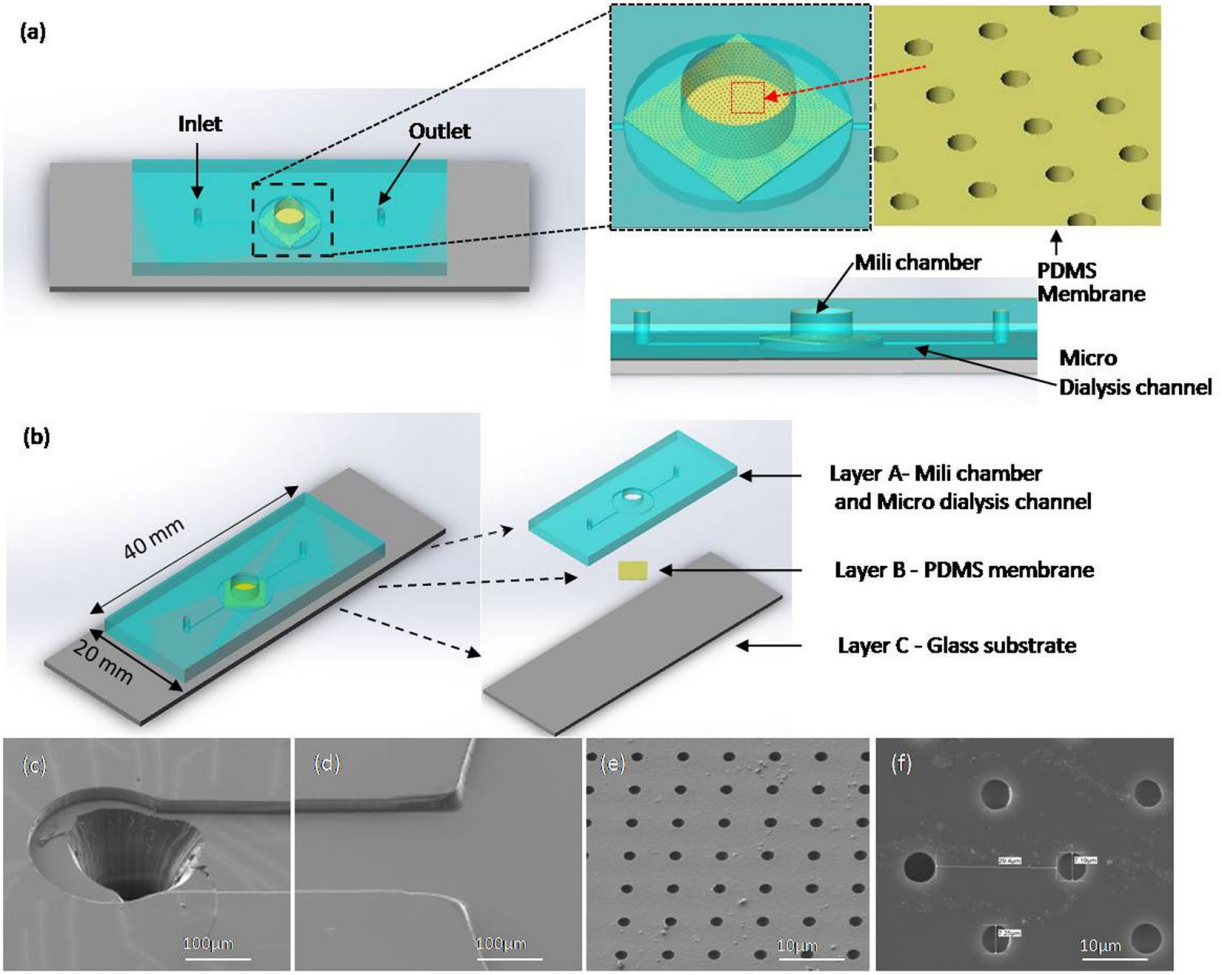
(**a**) The 3D schematic of the 3D-µdialysis chip, including the enlarged image of the openmili chamber which is the staining region dwelling on the top of PDMS porous membrane with an array of holes, and the cross section view illustrating the micro dialysis channel beneath the porous membrane. (**b**) Assembly of a 3D µdialysis chip, including layer A: PDMS chip containing mili chamber and micro dialysis channel, layer B: PDMS porous membrane, and layer C: Glass substrate. (**c**) and (**d**) are the SEM images of the PBS solution inlet for micro dialysis channel and the entrance to the bottom of the mili chamber region (beneath the porous membrane). (**e**) and (**f**) are the SEM images of the PDMS porous membrane with an array of holes (7 μm holes with 20 μm space).

Immunofluorescence was done to detect the characterization of circulating tumor cells (CTCs), regardless of the method of blood separation. In order to accelerate the staining process and avoid using a centrifuge to damage cells; a three-dimensional microwell dialysis (3D-μDialysis) chip with PDMS porous membrane was designed. There are three parts in construction of the chip. Figure 6(a) represents an overall 3D schematic view of the μDialysis chip while Fig. 6(b) deciphers a large PDMS milichamber residing on the top of a PDMS porous membrane constituting the whole device. The diameter of the hole is 7 mm, and height 3.0 mm and it acts as staining region for white blood cells and circulating tumor cells (CTCs). The thickness of the PDMS porous membrane is 40 μm and it bears an array of holes on its surfaces as depicted in Fig. 6(a). The diameter of milichamber is designed to be 7 μm to avoid cells losing from the holes because the size of white blood cells and CTCs are about 8~12 μm and 10~20 μm^23^ respectively. Under the PDMS porous membrane, a PDMS micro dialysis channel is formed and sandwiched between the porous membrane and a glass substrate. It is believed that after fully staining the cells, the residual fluorescence can gradually diffuses down to the bottom channel through the porous PDMS membrane and taken out by the bottom channel flow rapidly. Figure 6(c and d) represents the SEM images of inlet of the micro dialysis channel and staining region of the milichamber while Fig. 6(e and f) shows the SEM images of holes of the PDMS membrane.

### COMSOL simulation

The diffusion of dyes into the cells in 3D-μDialysis chip and in conventional model was simulated by using Comsol Multiphysics 5.1 to understand why staining efficiency is much improved in case of the 3D-μDialysis chip. The detailed simulation procedure was described in S1.

### Fabrication of the microfluidic chip

There are two parts of PDMS structure within the chip; a PDMS microdialysis channel with milichambers and a PDMS porous membrane and fabricated by using soft lithography technique. SU8 molds for micro dialysis channel and porous membrane were fabricated by using spin coating and baking procedures as described in Fig. S6. PDMS was etched by reactive ion etching and oxygen plasma was used to combine PDMS micro dialysis channel, PDMS porous membrane and glass, as depicted in Fig. S6.

### Cell culture

BT474 cell-line and HeLa cell-line is being utilized to spike into white blood cells to simulate the CTCs in patient’s blood. BT474 was cultured in medium contained RPMI, 10% foetal bovine serum (FBS), 1% Pen-Strep (P/S) at 37 °C and 5% carbon dioxide. HeLa cell-line was cultured in medium contained DMEM, 10% fetal bovine serum (FBS), 1% Pen-Strep (P/S) at 37 °C and 95% air with 5% carbon dioxide.

### Ethics statement and preparation of clinical samples

Currently, the 3D-μDialysis chip is also used to stain CTCs in the blood samples collected from Tapei Veterans General Hospital. Three clinical blood samples have been collected by following the ethical guide lines and protocols approved by Tapei Veterans General Hospital (Approval number is 2016-02-008CC). Informed consent was obtained from all three patients prior to involvement in the study. Upon collection, the red blood cells were removed from the whole blood first by using Leucosep™ tube, purchased from BIO-CHECK LABORATORIES LTD. In this study, gradient centrifugation was utilized to isolate red blood cells by Ficoll-Paque PLUS (GE Healthcare Life Sciences, Taiwan). This would result in lymphocytes layer containing CTCs after centrifugal process, and the CTCs cell retention rate is about 75–85%^24^.

### Immunofluorescence staining

Immunofluorescence dyes, Hoechst33258, CD45-PECy7 and EpCAM-FITC, are used in this study to identify the CTCs. Hoechst stains are part of a family of blue fluorescent dyes used to stain DNA. CD45-PECy7 is a protein stain while the CD45 antibody recognizes the human CD45 antigen, a tyrosine phosphatase also known as the leukocyte common antigen (LCA). Once the target cell conjugates with PE/Cy7 composite stain will excite certain light. EpCAM is a pan-epithelial differentiation antigen that is expressed on almost all carcinomas as 17–1 A(mAb) antigen while FITC is a fluorochrome with a molecular weight of 389 Da.

### Staining in 3D-μDialysis chip

In this study, three fluorescence dyes were used to identify the type of cells on the 3D-μDialysis chips for both the BT474 spiked white blood cell samples and clinical samples. First, Hoechst33258 (10 µg/ml) was utilized to stain the nuclear region, and then EpCAM-FITC (2.5 µL/ml) was used to stain the epithelial cell surface antibody of BT474 cells, HeLa cells or CTCs in clinical samples, and CD45-PECy7 (27.78 µL/ml) to stain the antibodies of white blood cells, respectively.

### Attachment staining, static/dynamic staining process

Hoechst33258 was used to stain the nuclear region of BT474 cells on 3D-μDialysis chip for the test and a syringe pump was used to inject PBS solution into the inlet of chip in 10 minutes (S2). In the zero flow rate the flow in the bottom microdialysis channel was shut off right after it is fully filled with PBS buffer solution. Since there is a continues concentration-gradient of fluorescence dyes between the top open chamber and the bottom micro dialysis channel, the residual dyes would be first either diffused (in the static flow condition) or dialyzed (in dynamic flow conditions) down to the bottom channel and then retained in the microdialysis channel (in the static flow) or washed away by PBS solution (in dynamic flows) depending on different microdialysis channel flow rates, respectively.

The staining results between traditional attachment staining and 3D-μDialysis staining were also compared. BT474 cell line was incubated in a flask at 37 °C and 95% air and 5% carbon dioxide for five days before test. Hoechst 33258 or EpCAM-FITC dyes were introduced into the flask with BT474 cells attached on the bottom surface in a concentration of 10 µg/ml, and the flask was kept in a dark environment at room temperature for 30 minutes. PBS solution was utilized to wash the residual fluorescence dye and the washing procedure was repeated thrice by pipette. CD45-PECy7 (27.78 µg/ml) was introduced into a tube with white blood cells and the tube was kept in a dark environment at room temperature for 30 minutes. PBS solution was utilized to wash the residual fluorescence dye and the washing procedure was repeated thrice by pipette. The results were utilized to compared with the 3D-μDialysis staining results at the same concentration of introduced fluorescence dyes.

### Optimization of flow rate and staining time for 3 fluorescence dyes

Different flow rates in the micro dialysis channel were performed to optimize the conditions for staining processes such as to reduce the staining duration and to use of minimal volume for staining procedure in 3D-μDialysis chip. Hence, the flow rate was optimized by setting it to be 0 ml/hr, 10 mL/hr, 15 ml/hr, and 20 ml/hr for 20 minutes on the 3D-μDialysis chip for 3 fluorescence dyes individually. In the later part of the experiment another 2 parameters such as 13 mL/hr, and 17 ml/hr was introduced for a precision optimization of process flow. Hoechst 3325 was utilized to stain the DNA in the nucleus region of HeLa cell line and white blood cell, CD45-PECy7 was introduced to stain white blood cells and EpCAM-FITC was used to identify HeLa cells in this experiment.

### Mixed continuous staining

In order to outline a procedure which would save time for using three fluorescence dyes staining together, after testing each dye individually, all the three dyes were originally considered to be mixed altogether and tested on the cells for simultaneously staining. However, because of its chemical nature to interact with the anitibodies on CD45-PECy7 and EpCAM-FITC, Hoechst33258 is unable to stain cells together with CD45-PECy7 and EpCAM-FITC. Therefore in the mixed dyes staining test, Hoechst33258 was used first to stain the nucleus of cells for 10 minutes, and then the mixture of CD45-PECy7 and EpCAM-FITC was introduced simultaneously for the next 25 minutes of staining.

### Staining of clinical sample

We collaborate with Taipei Veterans General Hospital for detection of stage IV colon cancer by using our fabricated 3D-μDialysis chip. Three cases have been received and treated with the above cited staining procedure by using 3D-μDialysis chip to identify the CTCs.

Hoechst33258 was used first to stain the nucleus of cells for 10 minutes, and then the mixture of CD45-PECy7 and EpCAM-FITC was introduced simultaneously for the next 25 minutes to stain with a flow rate of 0 ml/h for the first 10 minutes followed by a flow rate of 15 ml/h for the rest 15 minutes. As morphology of CTC doesn’t define clearly, it is assumed that particles stained with EpCAM^+^, Hoechst^+^ and CD45-PECy7^−^ are treated as CTCs^6^. Thus EpCAM^+^ based CellSearch system is applied in the present research to identify CTCs.

### Imaging

The fluorescence imaging was carried out by using normal optical fluorescence microscopes (Olympus IX71) by using the appropriate wavelength for different dyes. The excitation and emission wavelength were 380 and 488 nm for Hoechst 33258, 566 and 778 nm for CD45-PECy7, as well as 495 and 519 nm for EpCAM-FITC respectively. The exposure time of each sample for imaging was kept as 0.1 second.

### Cell viability assay

Cell viability assay was carried out by using propidium bromide (PI) dye to understand the role of centrifugation in case of conventional staining as this particular dye only enters into the ruptured cells and stain DNA. PI of 4 µmol/L was added into each of the samples with (conventional staining) or without (3D µdialysis staining) centrifugation. The automated cell counter (Moxi™ Z Mini Automated Cell Counter, ORFLO Technologies) was used to acquire the cell counts with various diameters.

### Statistical analysis

All the experiments were performed in triplicate and the data were represented with their corresponding relative standard deviations (RSD). The images were analyzed by image processing software, ImageJ to obtain the histogram of the fluorescence intensity on cells and backgrounds. ImageJ software transfers the original fluorescence images into grey scale images. In grey scale mode, light intensity was scaled numerically from 0 to 255 that means brighter part has more numbers. In this context the average signal light intensity was obtained by averaging the light intensities of 10 different cells while average background light intensity reflects the average of 10 different background areas. The values were further used to derive signal to noise (SNR) ratio by using the Equation 1. The higher SNR can lead to better recognition of cell’s morphology; hence we use SNR to be the important index to testify the staining efficiency.

1 $$SNR=\frac{Average\,signal\,light\,\mathrm{int}\,ensity}{Average\,background\,light\,\mathrm{int}\,ensity}$$

### Data availability

All data generated or analyzed during this study are included in this published article (and its Supplementary Information files).

## Electronic supplementary material

Dataset 1
