# Author Correction: Rapid Staining of Circulating Tumor Cells in Three-Dimensional Microwell Dialysis (3D-μDialysis) Chip

**DOI:** 10.1038/s41598-018-26548-5

**Published:** 2018-05-29

**Authors:** Wanying Cho, Rangadhar Pradhan, Hsin Ying Chen, Yi-Hsuan Weng, Hsueh Yao Chu, Fan-Gang Tseng, Chien-Ping Lin, Jeng-Kai Jiang

**Affiliations:** 1 Department of Engineering and System Science, Frontier Research Center on Fundamental and Applied Sciences of Matters, National Tsing-Hua University, Hsinchu, Taiwan, ROC; 2 Institute of NanoEngineering and MicroSystems, National Tsing-Hua University, Hsinchu, Taiwan, ROC; 3 Division of Colon and Rectal Surgery, Department of Surgery, Taipei Veterans General Hospital, Taipei, Taiwan, ROC; 4 Research Center for Applied Sciences, Academia Sinica, Taipei, Taiwan, ROC

Correction to: *Scientific Reports* 10.1038/s41598-017-09829-3, published online 12 September 2017

In the original version of this Article, there was an error in Affiliation 1 which was incorrectly listed as “Department of Engineering and System Science, National Tsing-Hua University, Hsinchu, Taiwan, ROC”. The correct affiliation is listed below:

“Department of Engineering and System Science, Frontier Research Center on Fundamental and Applied Sciences of Matters, National Tsing-Hua University, Hsinchu, Taiwan, ROC”

In addition, the original version of this Article omitted an affiliation for Fan-Gang Tseng. The correct affiliations are listed below:

“Department of Engineering and System Science, Frontier Research Center on Fundamental and Applied Sciences of Matters, National Tsing-Hua University, Hsinchu, Taiwan, ROC”

“Research Center for Applied Sciences, Academia Sinica, Taipei, Taiwan, ROC”

Finally, the Acknowledgements section in this Article was incomplete.

“The authors are thankful to the Ministry of Science and Technology, Taiwan to support the present study (Project Number: MOST-104-2221-E-007-072-MY3).”

now reads:

“The authors are thankful to VGHUST Joint Research Program and the Ministry of Science and Technology, Taiwan to support the present study (Project Number: MOST-104-2221-E-007-072-MY3).”

These errors have now been corrected in the HTML and PDF versions of this Article.

