# Supplementary material for: Rapid Staining of Circulating Tumor Cells in Three-Dimensional Microwell Dialysis (3D-μDialysis) Chip
Source: Sci Rep. 2017 Sep 12;7:11385. doi: 10.1038/s41598-017-09829-3 (PMC5595982; doi:10.1038/s41598-017-09829-3)
Supplement: Supplementary file 1 — Dataset 1 [file 41598_2017_9829_MOESM1_ESM.doc]

Supplementary Data

**Rapid Staining of Circulating Tumor Cells in Three-Dimensional Microwell Dialysis (3D-μDialysis) Chip**

Wanying Cho^a#^, Rangadhar Pradhan^a#^, Hsin Ying Chen^b^, Yi-Hsuan Weng^a^, Hsueh Yao Chu^a^, Fan-Gang Tseng^a*^, Chien-Ping Lin^c^, Jeng-Kai Jiang^c^

| (a) | 5^th^ min | 10^th^ min | 15^th^ min | 20^th^ min |
| --- | --- | --- | --- | --- |
| 0 ml/hr | 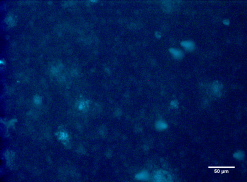 | 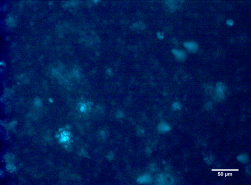 | 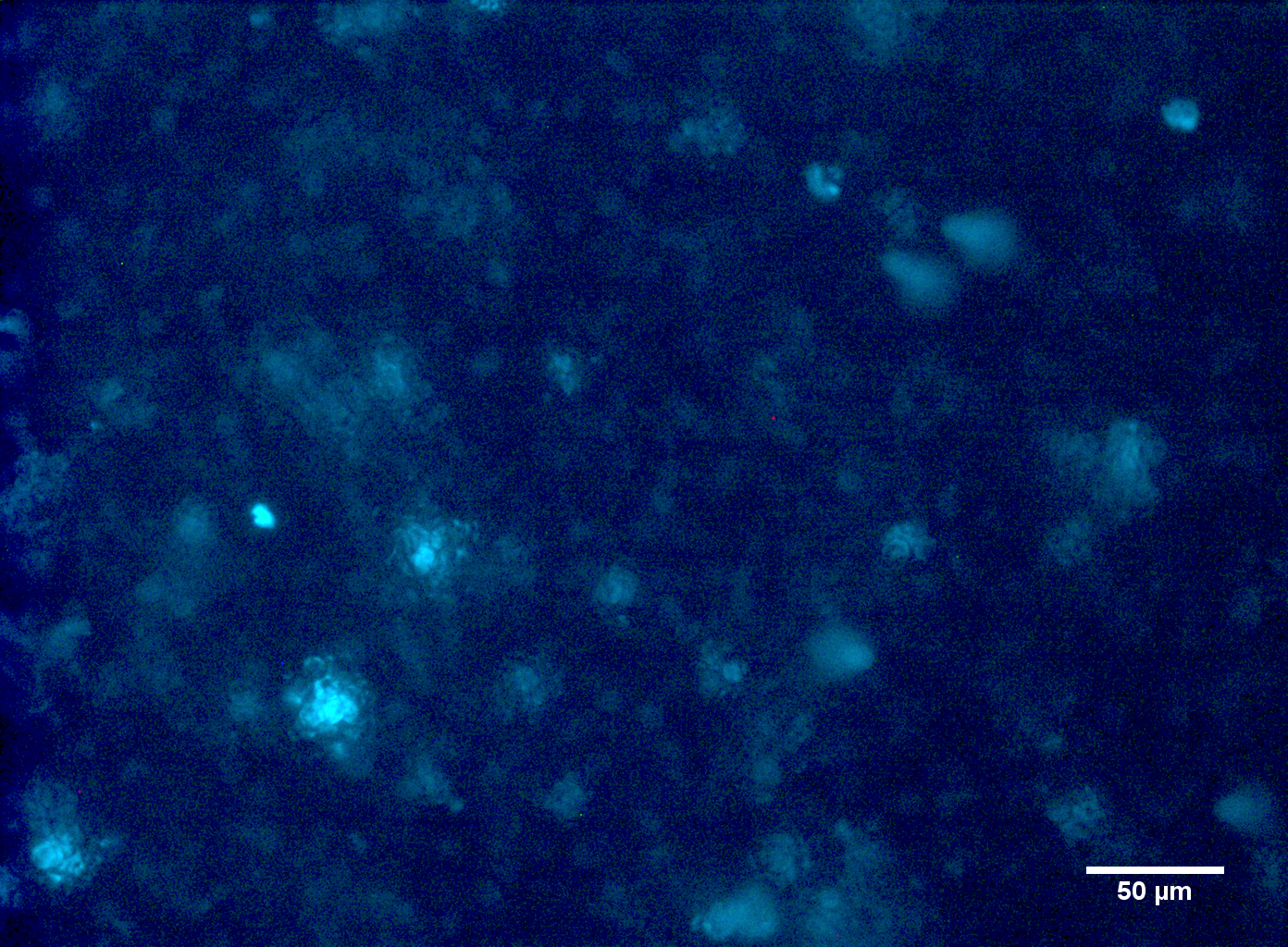 | 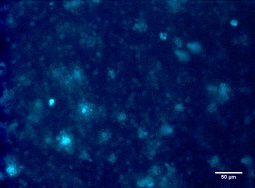 |
| 10 ml/hr | 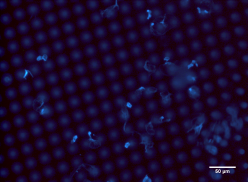 | 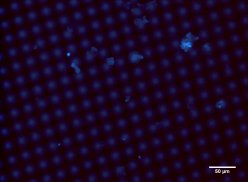 | 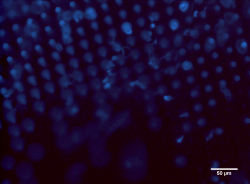 | 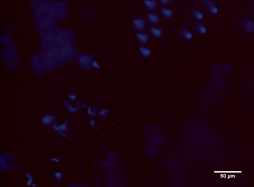 |
| 15 ml/hr | 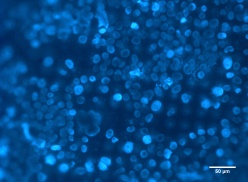 | 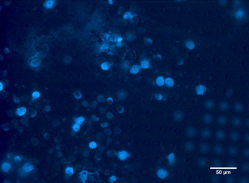 | 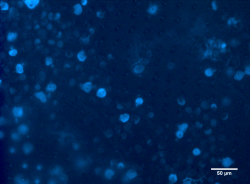 | 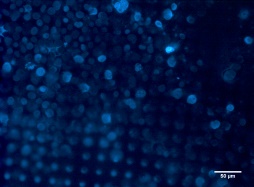 |
| 20 ml/hr | 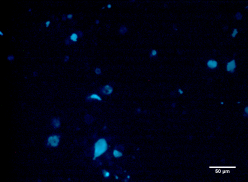 | 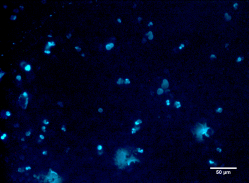 | 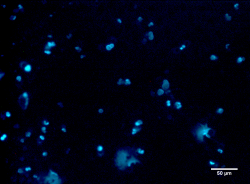 | 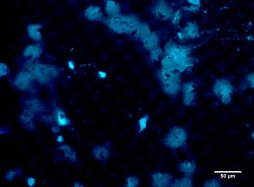 |

| (b) | 5^th^ min | 10^th^ min | 15^th^ min | 20 ^th^ min |
| --- | --- | --- | --- | --- |
| 0 ml/hr | 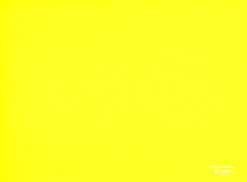 | 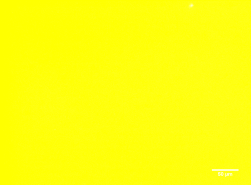 | 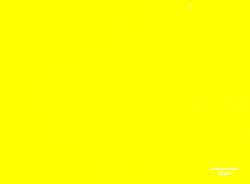 | 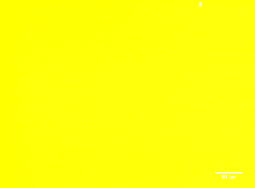 |
| 10 ml/hr | 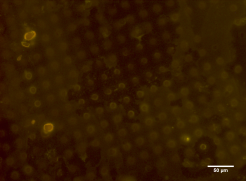 | 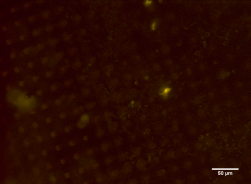 | 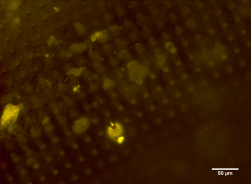 | 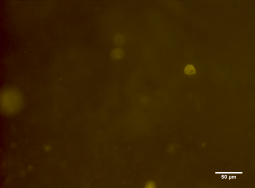 |
| 15 ml/hr | 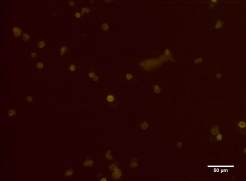 | 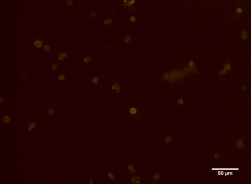 | 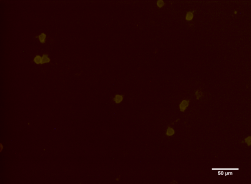 | 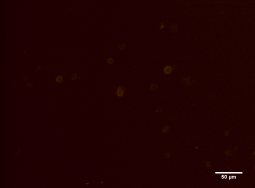 |
| 20 ml/hr | 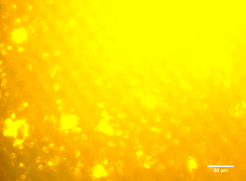 | 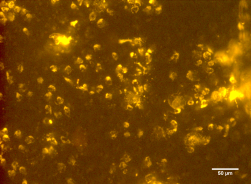 | 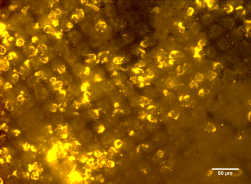 | 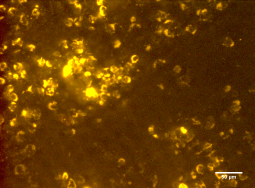 |

| (c) | 5^th^ min | 10^th^ min | 15^th^ min | 20^th^ min |
| --- | --- | --- | --- | --- |
| 0 ml/hr | 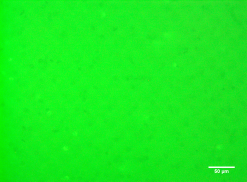 | 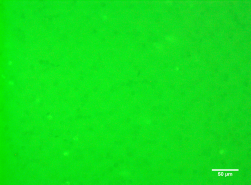 | 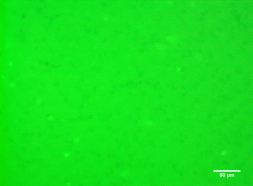 | 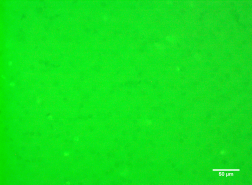 |
| 10 ml/hr | 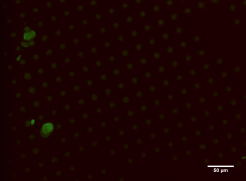 | 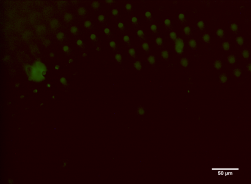 | 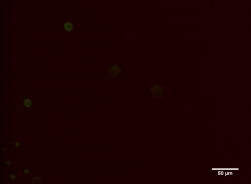 | 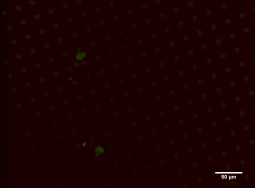 |
| 15 ml/hr | 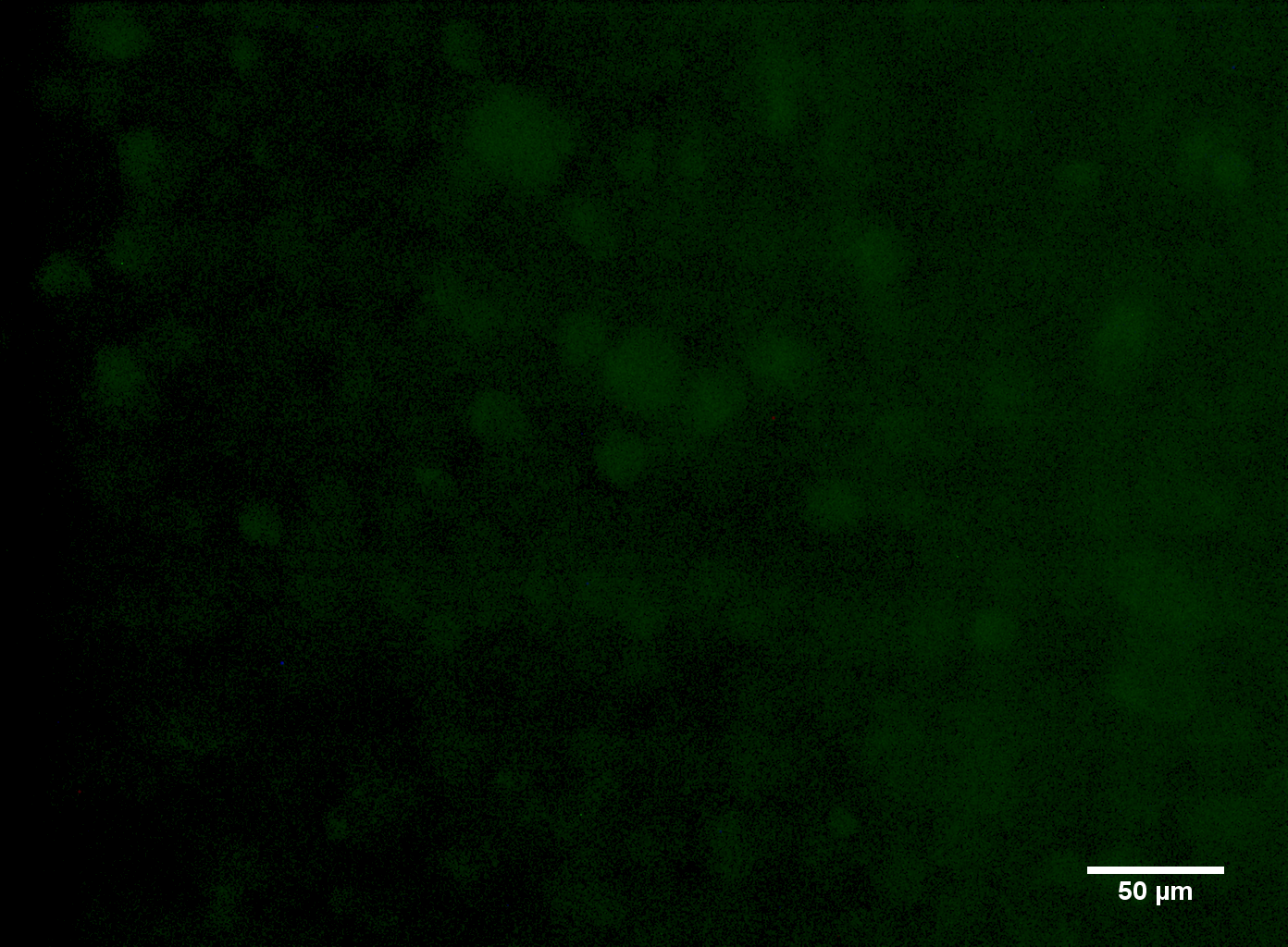 | 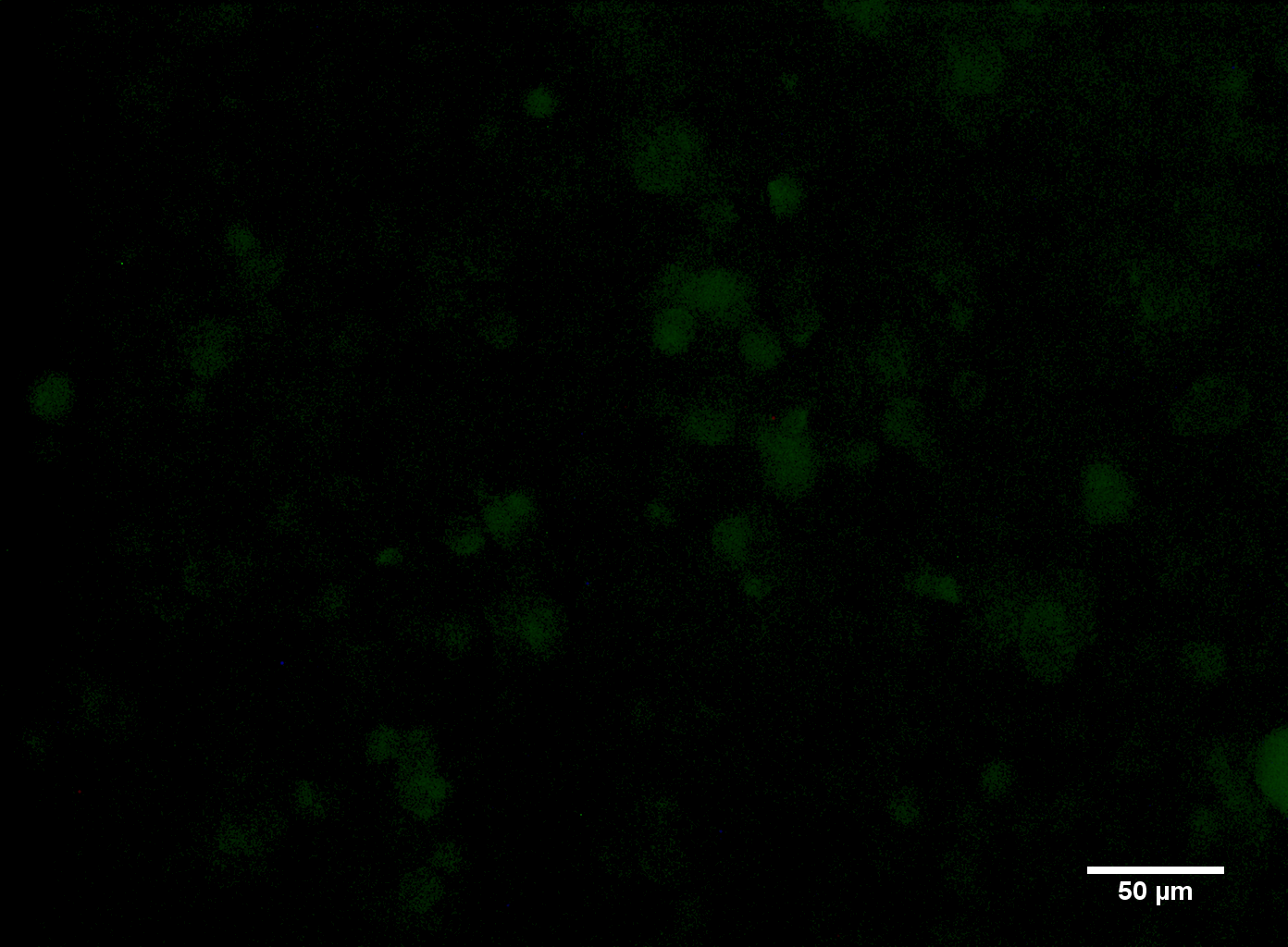 | 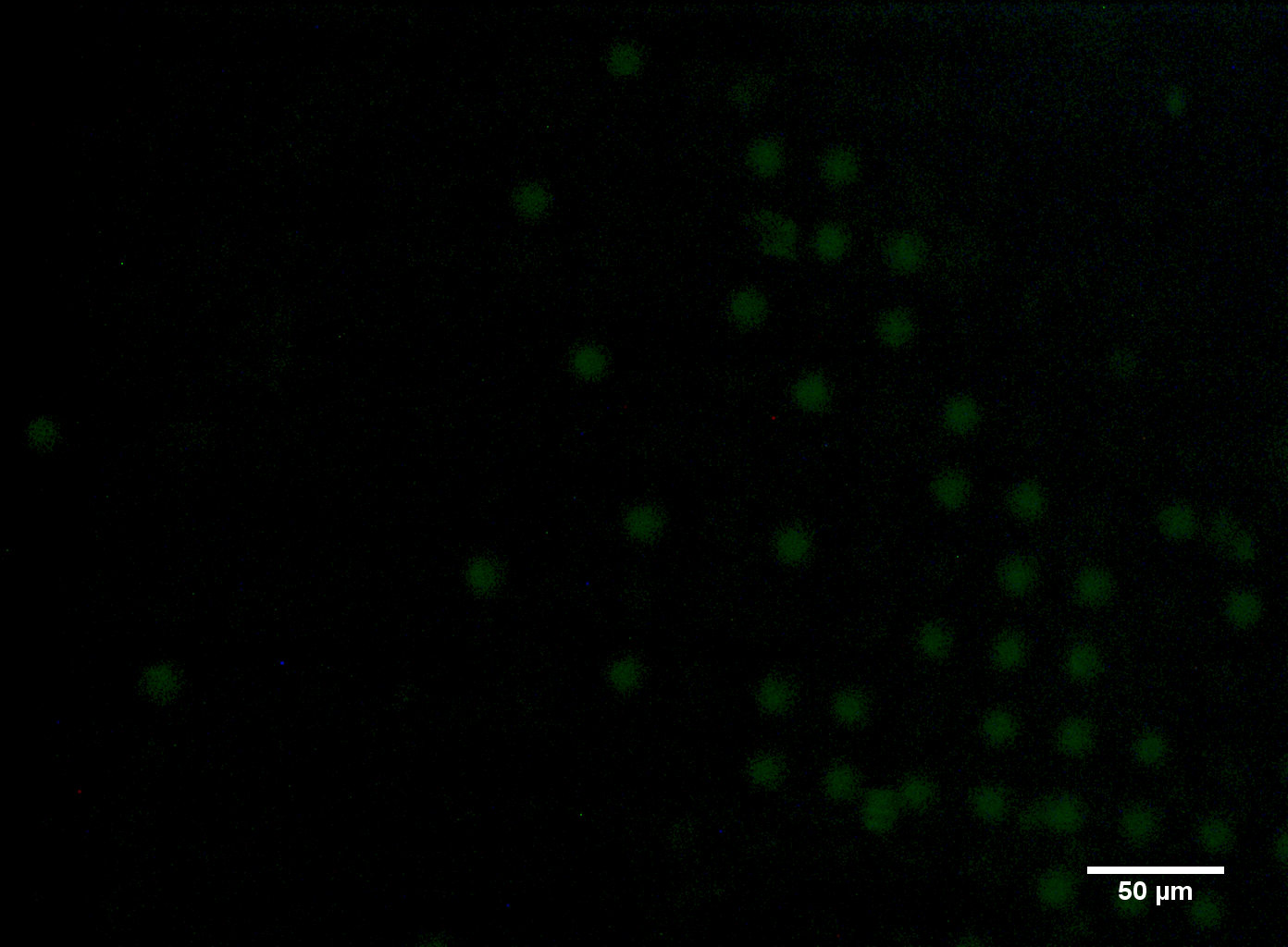 | 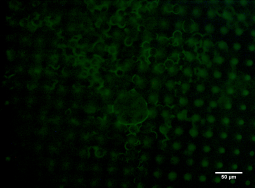 |
| 20 ml/hr | 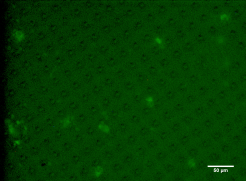 | 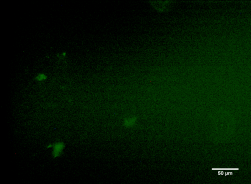 | 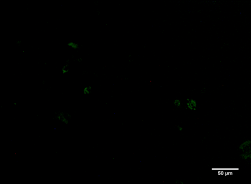 | 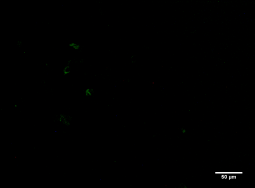 |

**Figure S1.** Three fluorescence dyes under different flow speeds of dynamic staining for 20 minutes. (a) Hoechst 33258 staining for white blood cells and Hela cell line (b) CD45-PECy7 staining for white blood cell, and (c) EpCAM-FITC staining for Hela cell line. Each scale bar is 50 µm.

**
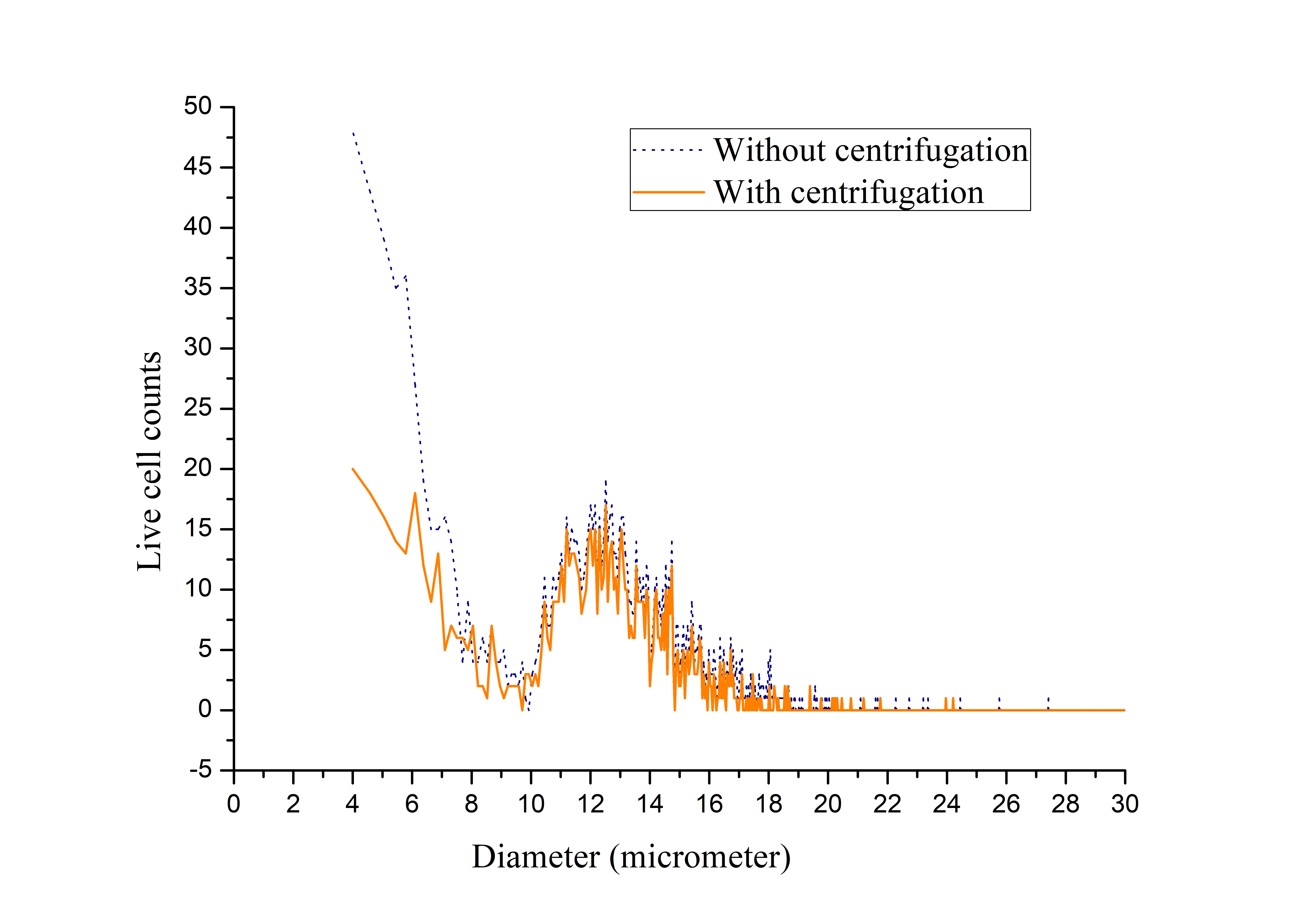
**

| (a) | Visible | PI |
| --- | --- | --- |
| Without centrifugation | 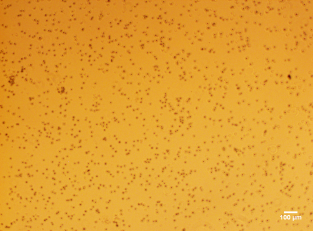 | 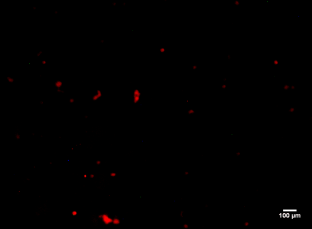 |
| With  centrifugation | 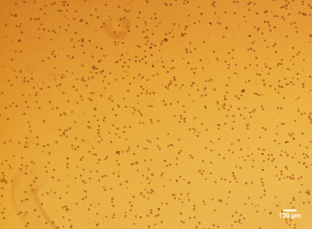 | 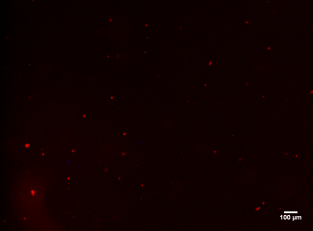 |

(b)

**Figure S2.** The cell viability assay as (a) represents a plot of the cell count versus cell diameter while (b) represents images of sample with or without centrifugation. (The Propidium Iodide enters into the ruptured cells and stains DNA represented as stained particles in the image. These particles are less in sample without centrifugation).

(a)

| 3^rd^ min | 5^th^ min | 7 ^th^ min | 10 ^th^ min |
| --- | --- | --- | --- |
| 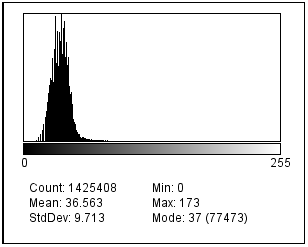 | 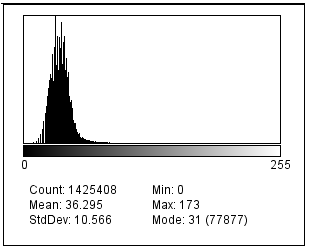 | 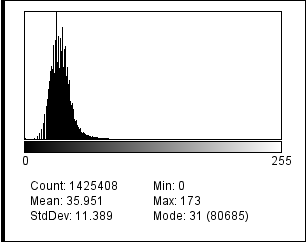 | 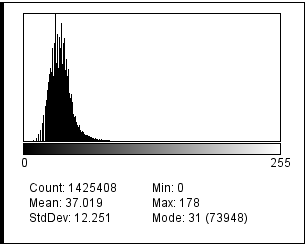 |
| 13 ^th^ min | 15 ^th^ min | 17 ^th^ min | 20 ^th^ min |
| 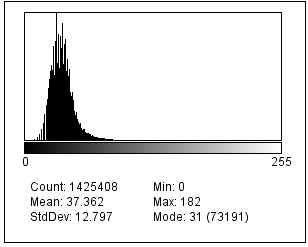 | 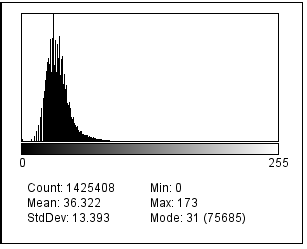 | 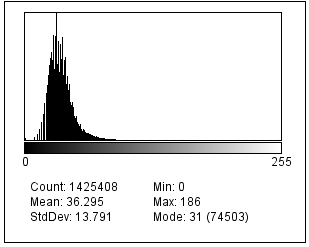 | 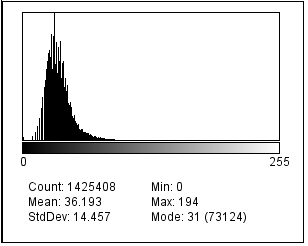 |

(b)

| 3^rd^ min | 5^th^ min | 7 ^th^ min | 10^th^ min |
| --- | --- | --- | --- |
| 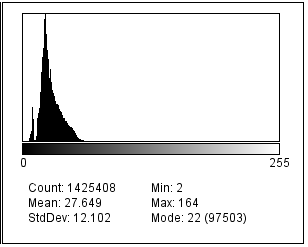 | 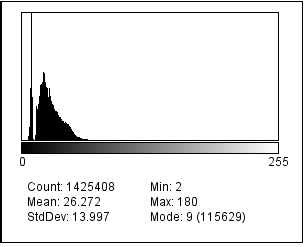 | 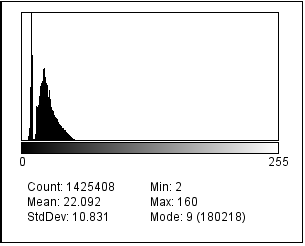 | 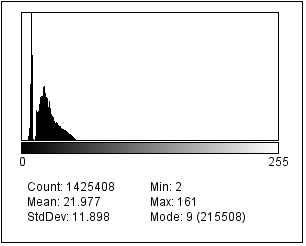 |
| 13^th^ min | 15^th^ min | 17^th^ min | 20^th^ min |
| 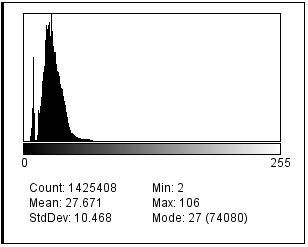 | 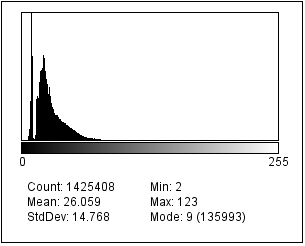 | 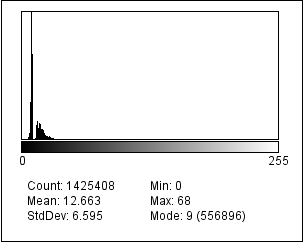 | 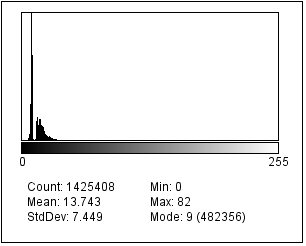 |

**Figure S3.** Histogram analysis of the 3D-μDialysis staining in (a) Static staining at a flow rate 0 ml/hr and (b) dynamic staining at a flow rate 10 ml/hr for Hoechst 33258 at the third, fifth, seventh, tenth, thirteenth, fifteenth, seventeenth, twentieth minute.

Add

Hoechst 33258

**30 min**

Removal

Process

**15 min**

Add

PECy7-CD45 & FITC-EpCAM

**30 min**

Removal

Process

**15 min**

**90 min**

**Conventional Staining Process**

Add

Hoechst 33258

**10 min**

Add

FITC-EpCAM (rest)

**15 min**

Add

PECy7-CD45

**10 min**

**35 min**

**3D-μDialysis Staining Process**

0 30 45 75

0 10 25

**Figure S4.** The time comparison between conventional staining process and 3D-µDialysis chip dynamic staining process.


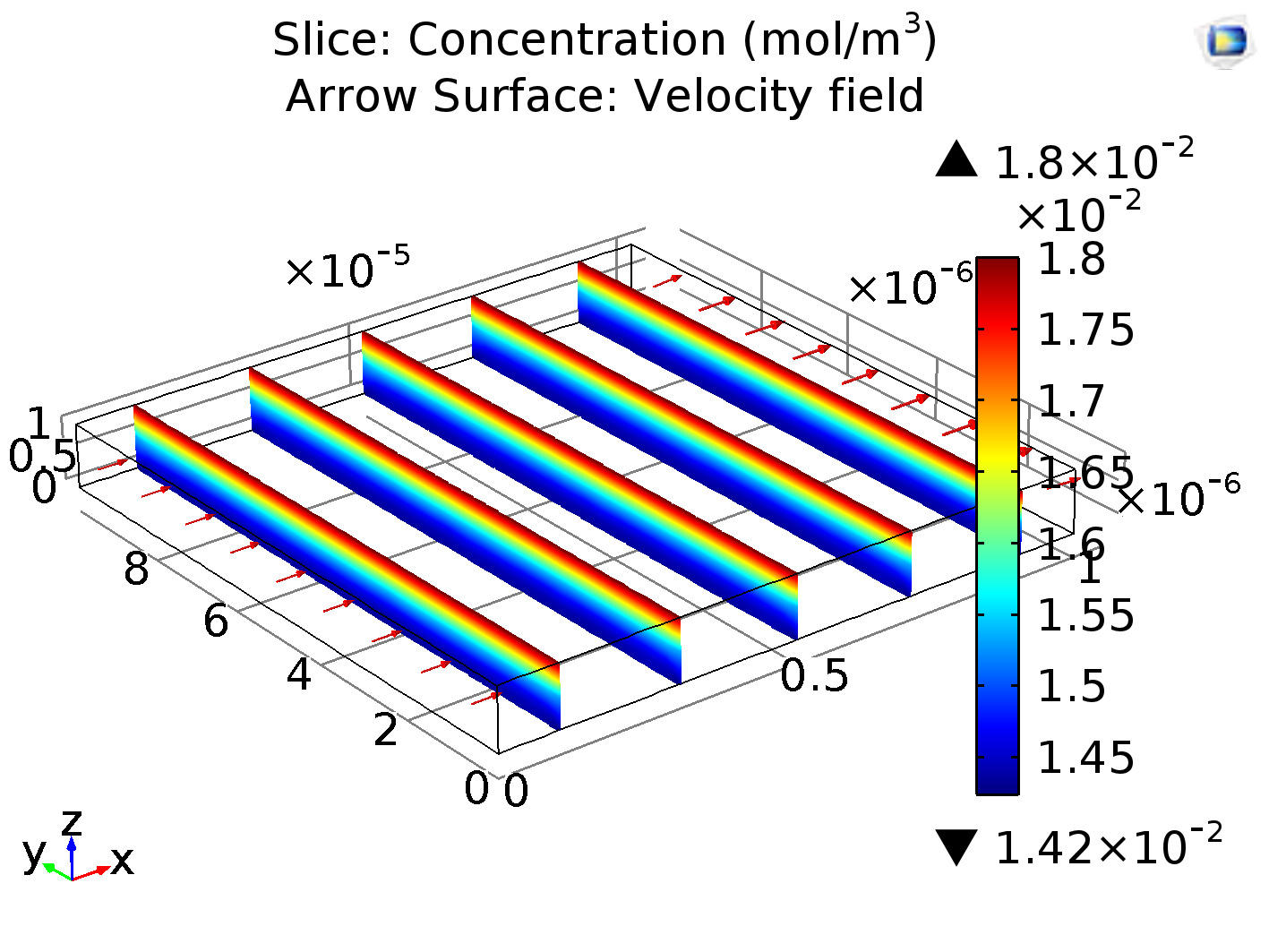

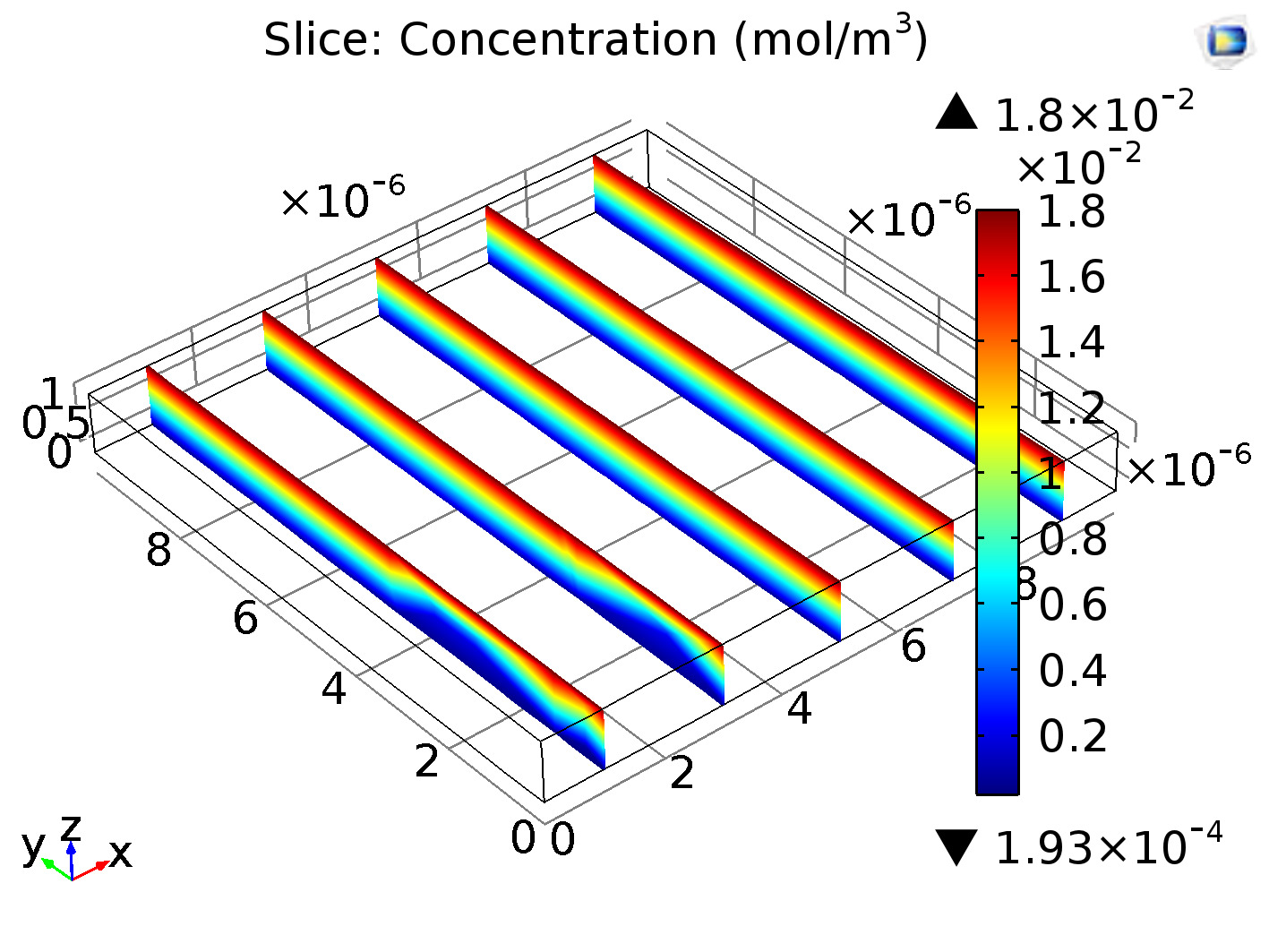


(a)

(b)

**Figure S5.** COMSOL results of concentration distribution (a) with flow and (b) without flow

**S1.**

A 3D block of 10×10×1 µm of dimensions, representing the cell in 3D-μDialysis chip was simulated to understand the diffusion of dye through the cell. The block was simulated for dynamic staining in a flow rate of 10 ml/h by using both the laminar flow and transport of diluted species modules. The same block was simulated by using the transport of diluted species module to represent static staining method. Both the simulation was carried out in a small scale to avoid the limitations of Comsol simulation. Both the simulation was carried out by using a dye of 0.018 mol/m^3^ concentration to understand the diffusion process.


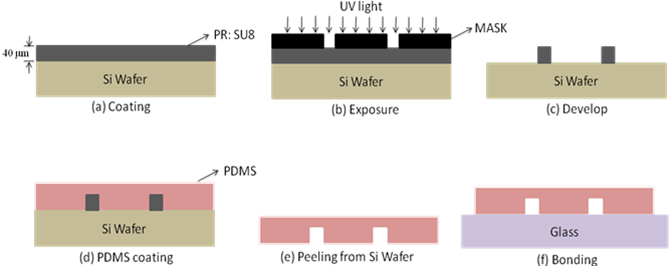


**Figure S6.** The fabrication process of 3D µdialysis chip

**S2.**

The time to diffuse layer L is calculated by using the following equation 1.

T = L^2^ / 2D (1)

Where, D is diffusion constant of fluorescein which is 500 μm^2^/sec and L is layer of diffusion.

Thus T = (790x790) μm^2^ / (2x500) μm^2^/sec

= 624.1 sec

= 10.4 min
